# Improvement of low temperature carbon combustion catalyst characteristic caused by mixing Bi_2_O_3_ with Tl_2_O_3_

**DOI:** 10.1038/s41598-021-88776-6

**Published:** 2021-05-05

**Authors:** Susumu Nakayama

**Affiliations:** Department of Applied Chemistry and Biotechnology, National Institute of Technology (KOSEN), Niihama College, 7-1 Yagumo-cho, Niihama-shi, Ehime, 792-8580 Japan

**Keywords:** Environmental sciences, Chemistry, Materials science

## Abstract

This study investigated the addition of various oxides to further improve the catalytic characteristics of Tl_2_O_3_, which offers a high carbon combustion catalytic capacity to lower the carbon combustion temperature of 660 °C by ~ 300 °C. Mixtures of carbon (2 wt%) with composite catalysts comprising 20 wt% Tl_2_O_3_–80wt% added oxide were analyzed using DSC. Bi_2_O_3_ offered the best improvement, where the exothermic peak temperatures for carbon combustion of carbon with various Tl_2_O_3_–*x* wt% Bi_2_O_3_ composites were lower than that of carbon with pure Tl_2_O_3_. Isothermal TG measurements were performed using a mixture of carbon and the Tl_2_O_3_‒95 wt% Bi_2_O_3_ composite catalyst, where a 2 wt% weight loss (i.e. removal of all carbon) was achieved above 230 °C. A porous alumina filter was coated with the composite catalyst and carbon was deposited on the filter surface. The filter was held at constant temperatures under air flow, which confirmed that carbon was completely removed at 230 °C. This study demonstrated the potential for using these composite catalysts in self-cleaning particulate filters to decompose and eliminate fine particulate matter and diesel particulate matter generated from steelworks, thermal power plants, and diesel vehicles simply using the heat of the exhaust gas in a factory flue-gas stack or vehicle muffler.

## Introduction

Carbon-based fine particulate matter with a diameter of less than 2.5 µm (PM2.5) is generated from steelworks and thermal power plants that consume large amounts of coal. The environmental pollution associated with PM2.5 has become a major social problem^1–3^. The technologies typically used to eliminate PM2.5 include filtering, where multiple filters of different fineness values are used^4,5^, and electrical removal, where positively or negatively charged fine particle droplets are sprayed^6,7^. However, these methods have serious drawbacks, including the need for regular cleaning and complex systems.

Diesel engines offer high energy efficiency and the suppression of carbon dioxide emission, but generate exhaust gas containing diesel particulate matter (DPM) associated with human health risks and environmental contamination^8,9^. In response, research has aimed to develop carbon combustion catalysts to eliminate DPM exhausted from diesel engines at lower temperatures than the current conventional catalysts^10–17^. For example, La_0.8_Cr_0.9_Li_0.1_O_3_^10^, CeO_2_–ZrO_2_–Bi_2_O_3_^11^, La_0.9_Rb_0.1_CoO_3_^13^, and CeO_2_/Pr_4.8_Bi_1.2_O_11_^14^ catalysts have been reported. Exhaust gas from diesel cars can be controlled via the collection and elimination of DPM using a filter, where an oxidation catalyst is generally used. This technology has also been considered for the removal of PM2.5 originating from steelworks and thermal power plants.

**Figure 1:**
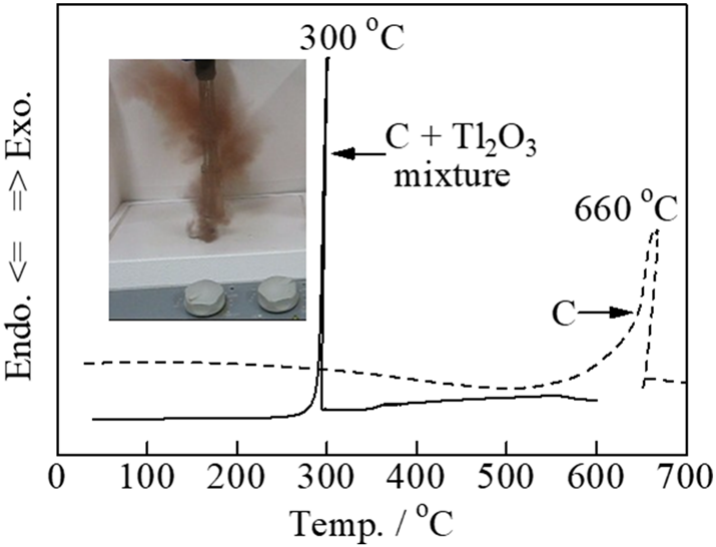
DSC curves of carbon black (5 wt%) mixed with a pure Tl_2_O_3_ catalyst. Inset is a digital photograph of explosive carbon combustion.

**Figure 2:**
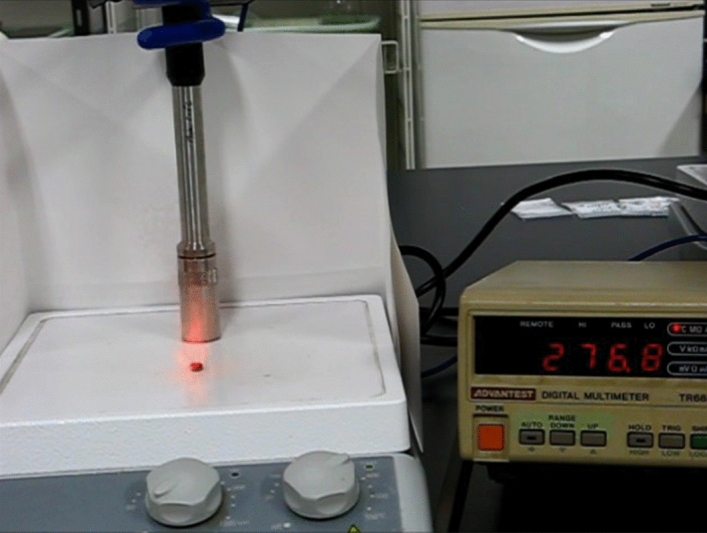
(Video Number—1) Video showing explosive carbon combustion with sparks.

Although the combustion temperature for pure carbon has been determined using differential scanning calorimetry (DSC) as 660 °C, this can be reduced to ~ 500 ºC by adding copper oxide to the carbon^18^. The combustion temperature may be reduced by a further 50 °C when a complex oxide with rare-earth elements are used^19^. Specifically, a previous study found that the addition of yttrium-manganate led to an exothermic DSC peak at ~ 430 °C, which resulted in good carbon combustion properties^20^. In addition, a previous report on Tl_2_O_3_ observed explosive carbon combustion at ~ 300 °C when 5% or more carbon was mixed with thallium (III) oxide (Tl_2_O_3_) (**Fig. ****1**) ^21^. The video of explosive carbon combustion with sparks can be found in **Fig. ****2**** (Video Number—1**). It should be noted that Tl^III^_2_O_3_ is not poisonous, unlike Tl^I^_2_SO_4_ and Tl^I^NO_3_. The application of Tl_2_O_3_ alone is not realistic because thallium is a rare and expensive metal. Thus, the development of Tl_2_O_3_ composites with other metal oxides is important.

This study aimed to composite Tl_2_O_3_ with other metal oxides that can supply oxygen in the lattice. The composites were evaluated based on their ability to maintain and improve the catalytic properties of Tl_2_O_3_ for low-temperature carbon combustion while reducing the amount of Tl_2_O in the catalyst. Furthermore, the collection of PM2.5 and DPM from exhaust gas was explored using porous ceramic filters coated with the catalytic composites for low-temperature carbon combustion. The filters show promise for applications in the flue-gas stacks of factories or on the muffler of cars. The performance of the self-cleaning type particulate filter for the collection of PM2.5 and DPM was demonstrated experimentally, where the carbon can subsequently be decomposed and removed simply due to the heat of the exhaust gas.

## Results and discussion

### Carbon combustion characteristics of the Tl_2_O_3_–80 wt% added oxide

**Figure 3:**
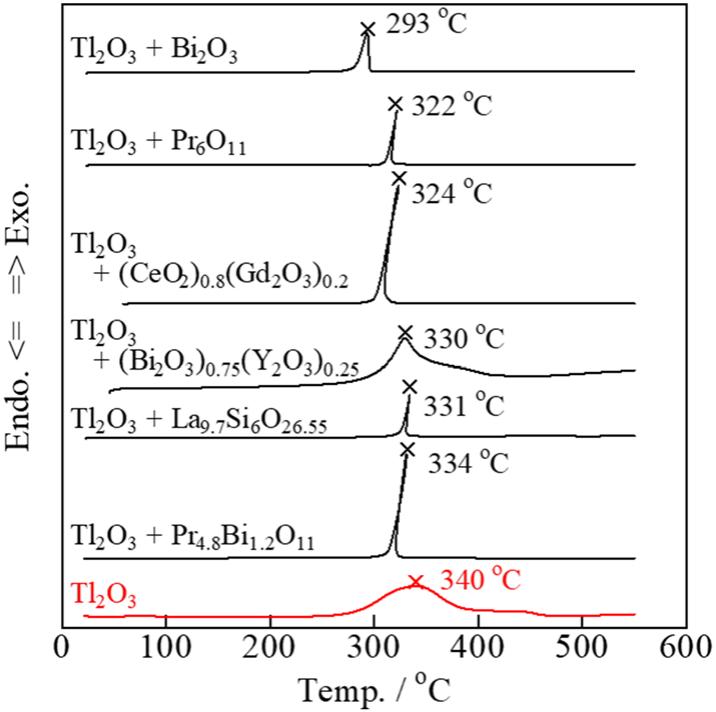
DSC curves of carbon black (2 wt%) mixed with the various Tl_2_O_3_‒80 wt% added oxide composite catalysts.

**Figure 4:**
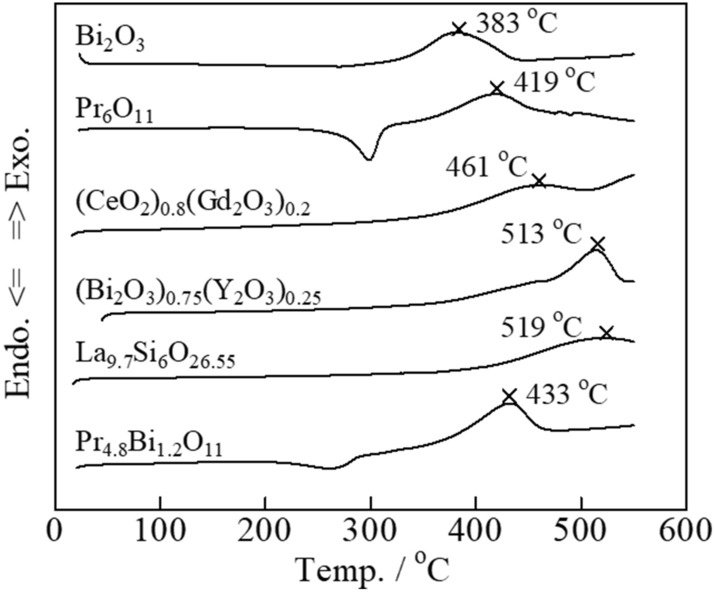
DSC curves of carbon black (2 wt%) mixed with the various oxide catalysts.

**Figure 5:**
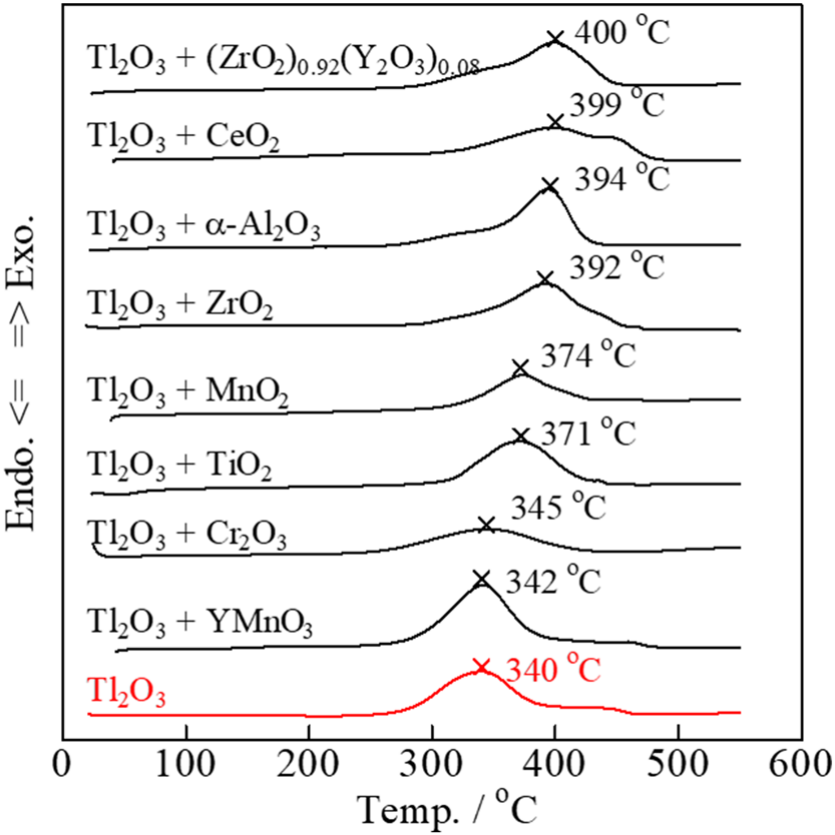
DSC curves of carbon black (2 wt%) mixed with the various Tl_2_O_3_‒80 wt% added oxide composite catalysts.

Tl_2_O_3_ offers excellent carbon combustion properties, and was mixed with both general single oxides, namely CeO_2_, α-Al_2_O_3_, ZrO_2_, TiO_2_, Bi_2_O_3_, Pr_6_O_11_, Cr_2_O_3_, and MnO_2_, and complex oxides with ionic conductivity, namely (Bi_2_O_3_)_0.75_(Y_2_O_3_)_0.25_, (CeO_2_)_0.8_(Gd_2_O_3_)_0.2_, La_9.7_Si_6_O_26.55_, and Pr_4.8_Bi_1.2_O_11_. Further, YMnO_3_ was also investigated due to its promising catalytic properties for carbon combustion. The carbon combustion properties of the mixed composite catalysts were improved compared to that of a pure Tl_2_O_3_ catalyst when added to 2 wt% carbon (**Fig. ****3**). Specifically, the six oxides that led to improved carbon combustion were Bi_2_O_3_, Pr_6_O_11_, (CeO_2_)_0.8_(Gd_2_O_3_)_0.2_, (Bi_2_O_3_)_0.75_(Y_2_O_3_)_0.25_, La_9.7_Si_6_O_26.55_, and Pr_4.8_Bi_1.2_O_11_, where Bi_2_O_3_ had the largest effect. For comparison, the carbon combustion properties of the Bi_2_O_3_, Pr_6_O_11_, (CeO_2_)_0.8_(Gd_2_O_3_)_0.2_, (Bi_2_O_3_)_0.75_(Y_2_O_3_)_0.25_, La_9.7_Si_6_O_26.55_, and Pr_4.8_Bi_1.2_O_11_ catalysts with added to 2 wt% carbon are shown in **Fig. ****4**. The carbon combustion temperature for all samples was higher than 380 °C. However, the remaining eight oxides did not lead to improved carbon combustion properties (2 wt% carbon), namely CeO_2_, α-Al_2_O_3_, ZrO_2_, MnO_2_, TiO_2_, Cr_2_O_3_, (ZrO_2_)_0.92_(Y_2_O_3_)_0.08_, and YMnO_3_, where the carbon combustion performance was reduced compared to that of pure Tl_2_O_3_ (**Fig. ****5**).

### Carbon combustion characteristics of the Tl_2_O_3_ ‒Bi_2_O_3_ system

#### *Carbon combustion characteristics of Tl*_*2*_*O*_*3*_*‒x wt% Bi*_*2*_*O*_*3*_* (x* = *5‒95)*

**Figure 6:**
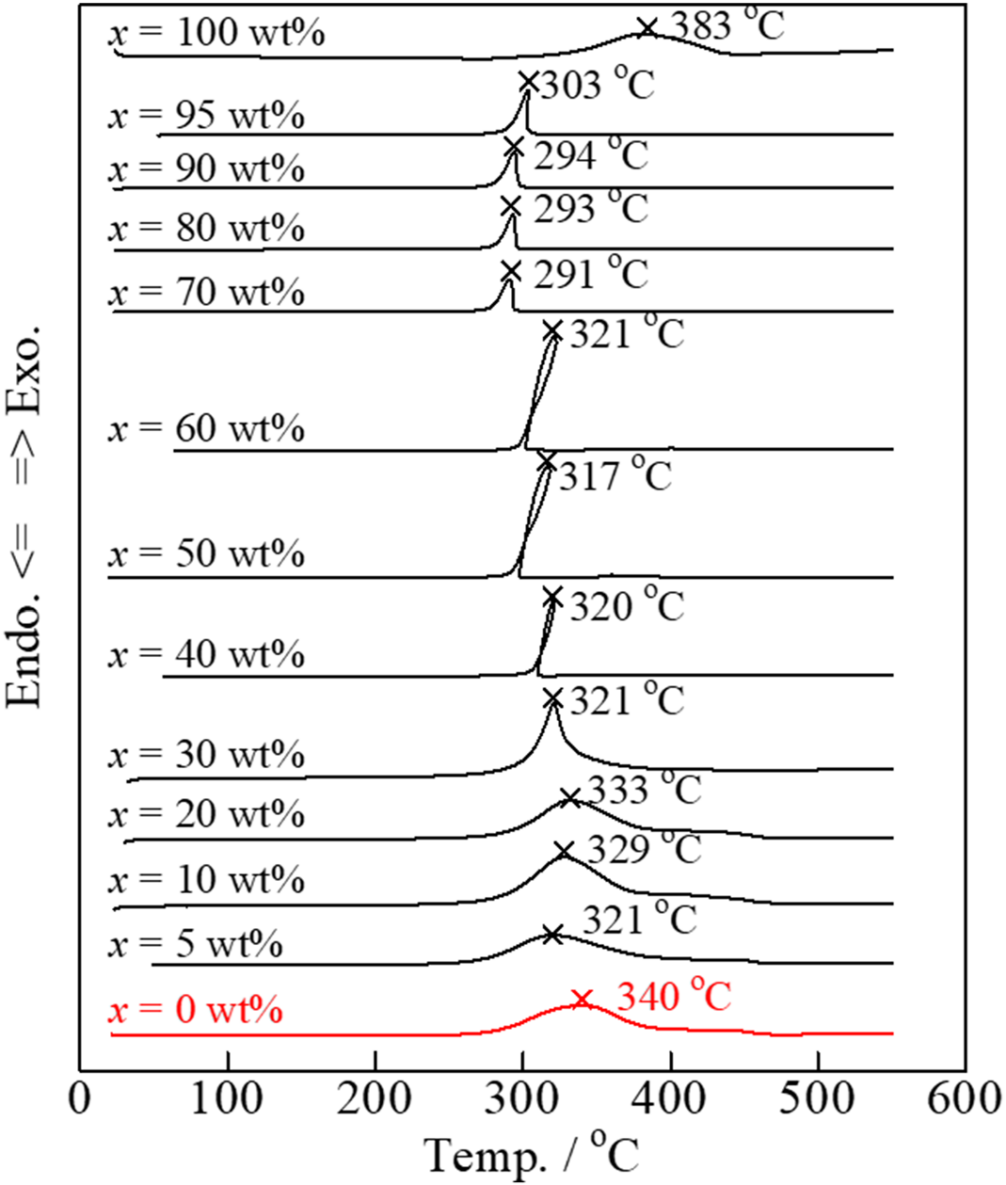
DSC curves of carbon black (2 wt%) mixed with the various Tl_2_O_3_‒* x* wt% Bi_2_O_3_ (*x* = 0–100) composite catalysts.

The addition of Bi_2_O_3_ led to the best carbon combustion properties (2 wt% carbon) among the various oxides added to Tl_2_O_3_. Thus, the relationship between the proportion of Bi_2_O_3_ in the composite catalyst and the carbon combustion properties was investigated. DSC analysis of the Tl_2_O_3_‒*x* wt% Bi_2_O_3_ (*x* = 0‒100) composite catalyst mixed with 2 wt% carbon was compared to Tl_2_O_3_‒*x* wt% Bi_2_O_3_ (*x* = 0‒100) with no added carbon to determine the carbon combustion performance (**Fig. ****6**). The DSC exothermal peaks of carbon combustion (2 wt% carbon) with pure Tl_2_O_3_ and Bi_2_O_3_ catalysts were observed at 340 and 383 °C, respectively. All of the Tl_2_O_3_‒*x* wt% Bi_2_O_3_ composite catalysts exhibited lower exothermal peak temperatures for carbon combustion compared to that of pure Tl_2_O_3_. In particular, the samples with *x* values of 70, 80, and 90 wt% exhibited exothermal peak temperatures during carbon combustion (2 wt% carbon) of 300 °C or less, which was indicative of excellent properties.

#### *Carbon combustion characteristics of thermal-treated Tl*_*2*_*O*_*3*_*‒95 wt% Bi*_*2*_*O*_*3*_

**Figure 7:**
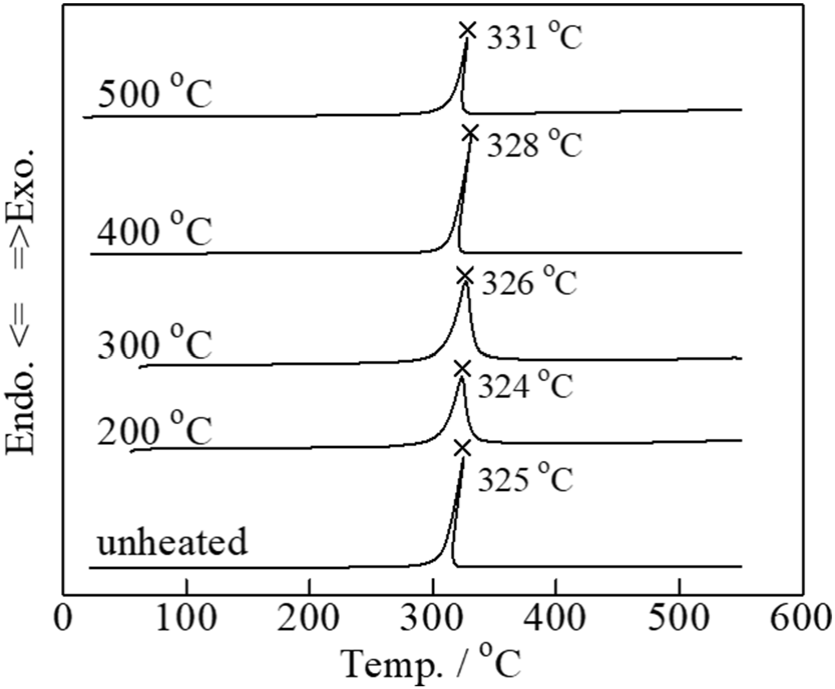
DSC curves of carbon black (2 wt%) mixed with Tl_2_O_3_‒95 wt%Bi_2_O_3_ and either left unheated or heat-treated at 200, 300, 400, and 500 °C for 2 h.

**Figure 8:**
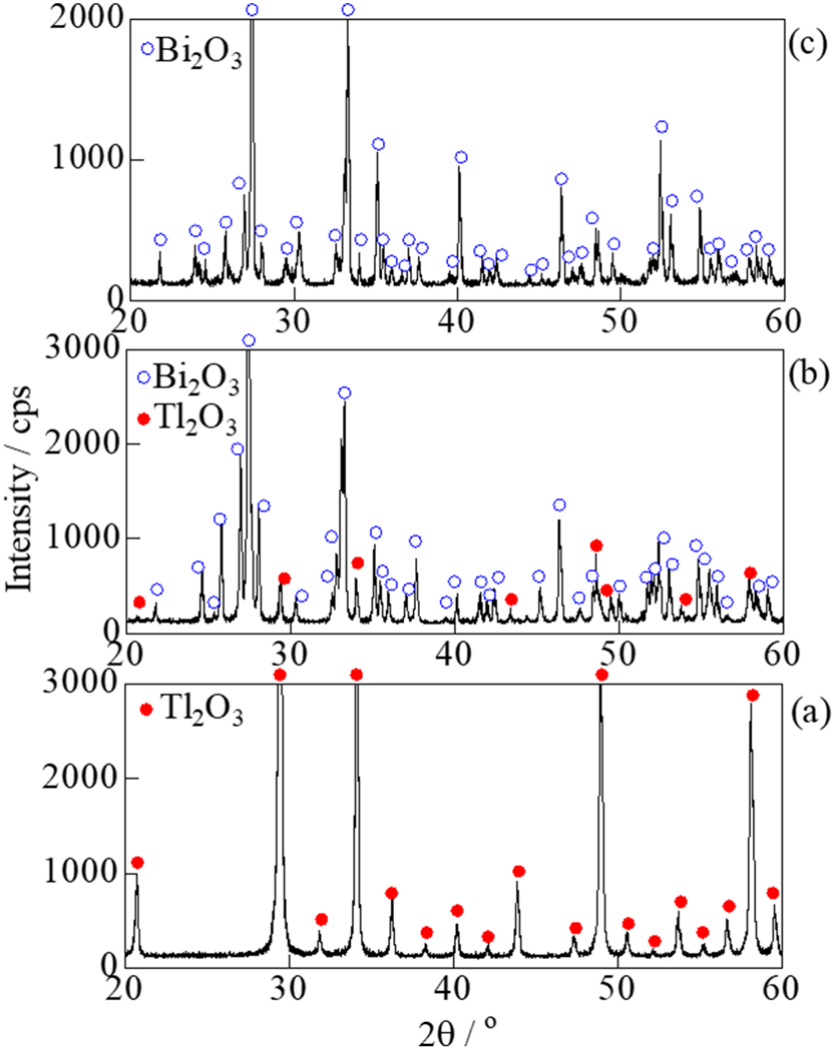
XRD analysis of (a) Tl_2_O_3_, (b) Tl_2_O_3_‒95 wt% Bi_2_O_3_, and (c) Bi_2_O_3_ after heat-treatment at 500 ℃ for 2 h.

Applications of carbon combustion catalysts often involve environments associated with the exposure to temperatures of ~ 500 °C. Carbon (2 wt%) was mixed with the Tl_2_O_3_‒95 wt % Bi_2_O_3_ catalyst, and was either left unheated or heat-treated at 200, 300, 400, and 500 °C. DSC analysis revealed minimal changes in the temperature of the exothermal peaks due to carbon combustion (**Fig. ****7**). Thus, heat treatments of 500 °C or below had a minimal effect on the carbon combustion properties of Tl_2_O_3_‒95 wt% Bi_2_O_3_. The formation of new phases in addition to Tl_2_O_3_ and Bi_2_O_3_ was evaluated during XRD analysis of the Tl_2_O_3_‒95 wt% Bi_2_O_3_ catalyst after heating at 500 °C (**Fig. ****8**). All of the XRD peaks observed at 2θ values of 20° to 60° were attributed to Tl_2_O_3_ and Bi_2_O_3_, thus the formation of complex oxides (e.g. Tl_3_BiO_3_) was not observed.

#### *Carbon combustion mechanism of the Tl*_*2*_*O*_*3*_*‒Bi*_*2*_*O*_*3*_* system*

**Figure 9:**
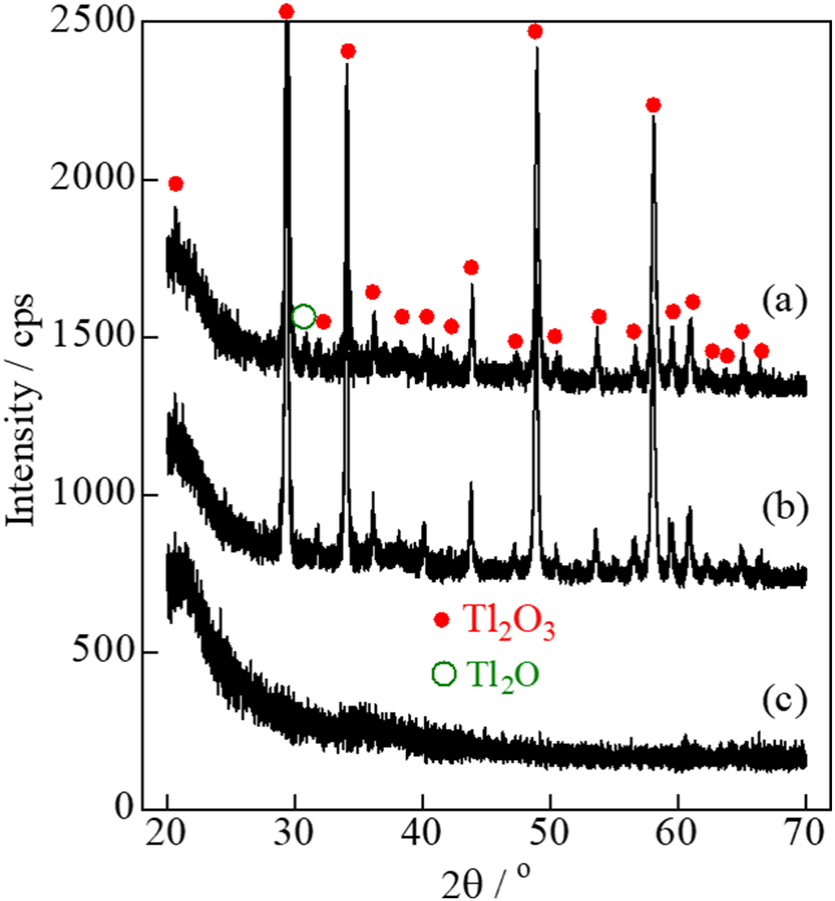
XRD analysis of the surface of porcelain dish. (a) Brown residue after reacting carbon black (5 wt%) mixed with Tl_2_O_3_ explosively at 300 ℃, (b) thermal-treated product after further heating of the brown residue in air at 300 ℃, and (c) an unused porcelain dish.

**Figure 10:**
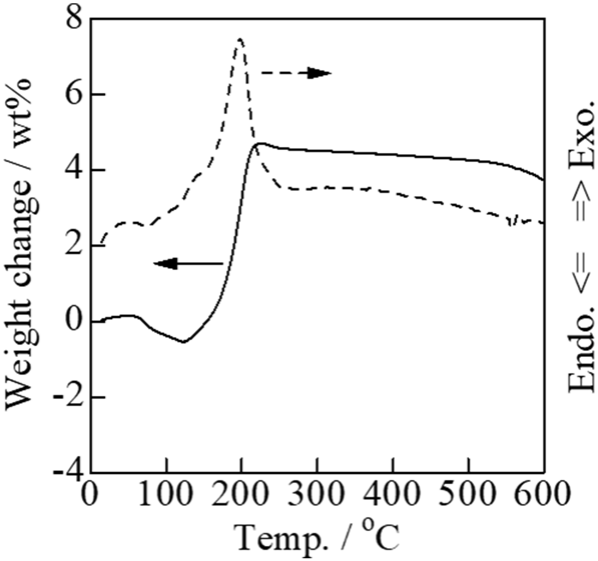
TG–DTA curves of Tl_2_O.

Thallium(III) oxide (Tl_2_O_3_) powder containing 5 wt%-carbon was placed on a porcelain dish (Nikkato Co., CW-1). When the mixture was gradually heated, explosive reaction (combustion) occurred around 300 ℃ and the reaction products were scattered (**Fig. ****1**). A very small amount of brown residue remained on the porcelain dish was subjected to the XRD measurement. The XRD pattern measured immediately after the explosive reaction showed a weak peak attributable to Tl_2_O around 2*θ* = 31° in addition to peaks attributed to Tl_2_O_3_ (**Fig. ****9****(a)**), whereas the peak attributable to Tl_2_O disappeared in the XRD pattern measured after further heating of the residue in air at 300 ℃ (**Fig. ****9****(b)**). As generally known, porcelain dish itself shows no diffraction peak (**Fig. ****9****(c)**). In order to investigate the cause of this disappearance, the TG–DTA measurement was performed for Tl_2_O (Kojundo Chemical Lab. Co., Ltd., 98% purity) in an air stream. **Figure ****10** shows an exothermic peak with a maximum at 200 ℃ resulting in an increase in weight which corresponds to the formation of Tl_2_O_3_ by the oxidation of Tl_2_O. In fact, it was confirmed that the sample obtained by igniting the Tl_2_O powder at 200 ℃ in a platinum crucible exhibited the XRD pattern of Tl_2_O_3_ and no peaks attributable to Tl_2_O. From these results, the carbon combustion mechanism on Tl_2_O_3_ can be presumed as follows:

Tl_2_O_3_ + 2C → 2 Tl_2_O + 2CO.

2CO + O_2_ → 2CO_2_.

Tl_2_O + O_2_ → Tl_2_O_3_.

At first, the lattice oxide ions in Tl_2_O_3_ are released for the oxidation of carbon to form CO and Tl_2_O. Then CO molecules immediately react with the surrounding oxygen at elevated temperature to give CO_2_ molecules. The Tl_2_O is also oxidized to Tl_2_O_3_ by the oxygen in air at 200 ℃ or above (300 ℃ in the present experimental condition) as shown in **Fig. ****10**. Thus, Tl_2_O_3_ functions as a combustion catalyst for carbon.

**Figure 11:**
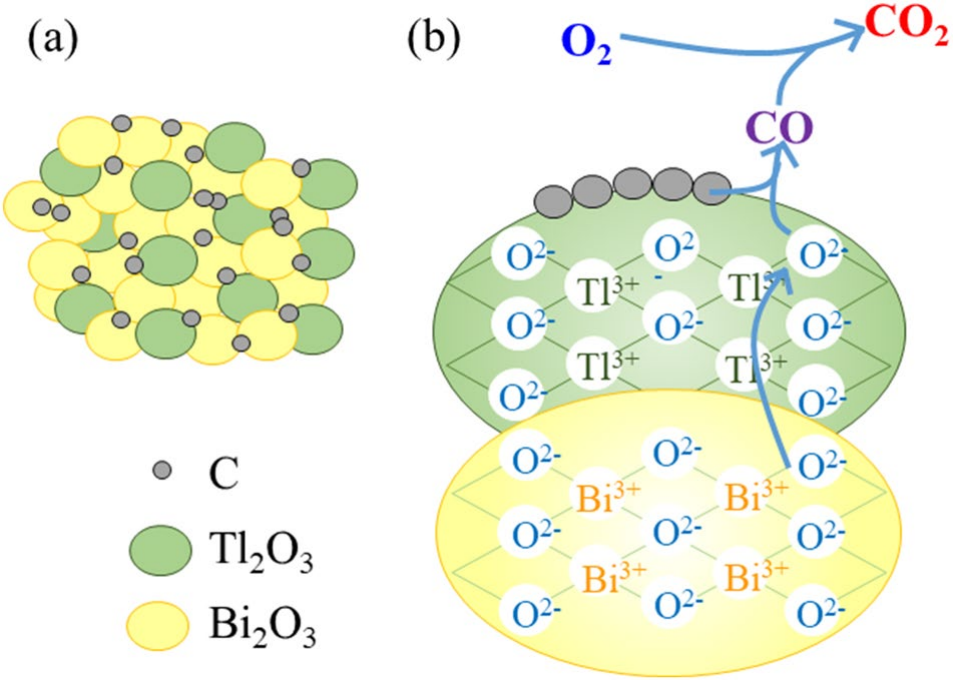
Schematic illustrations of (a) the carbon and Tl_2_O_3_‒Bi_2_O_3_ mixture and (b) the Tl_2_O_3_-modified Bi_2_O_3_-based carbon combustion catalyst and its oxygen transfer mechanism during carbon oxidation. The particle size of carbon is 14 nm (manufacturer data), and of Tl_2_O_3_ and Bi_2_O_3_ is 0.5 to 3 μm (SEM observations).

**Figure 12:**
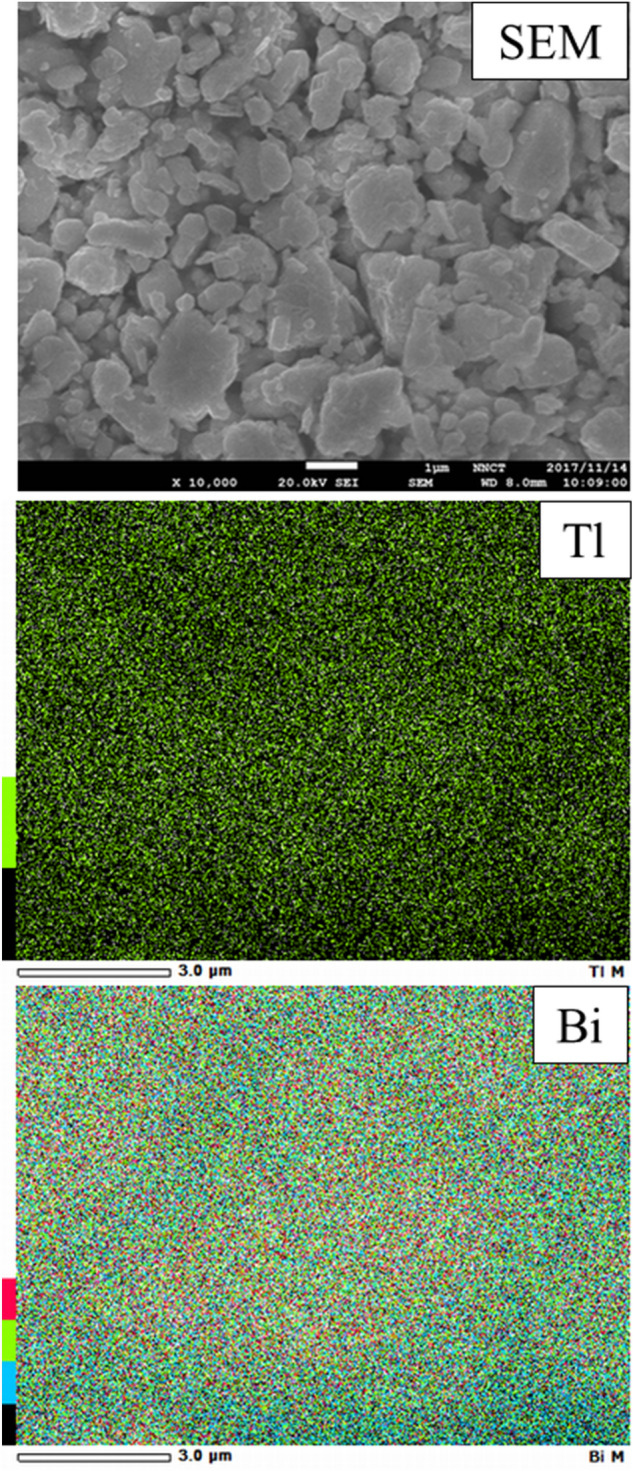
SEM image and EDS elemental mapping images of the Tl_2_O_3_‒95 wt% Bi_2_O_3_ composite catalyst.

The carbon combustion mechanism of the Tl_2_O_3_‒Bi_2_O_3_ system was proposed. Although the system comprised a smaller proportion of Tl_2_O_3_ compared to Bi_2_O_3_, the lattice oxygen in Tl_2_O_3_ was used for carbon combustion. The lattice defects of oxygen in Tl_2_O_3_ caused by carbon combustion were immediately recovered by the lattice oxygen in Bi_2_O_3_ (**Fig. ****11**). Therefore, the catalytic functioning for carbon combustion was continuously maintained in Tl_2_O_3_. Furthermore, the high oxygen desorption properties of Bi_2_O_3_ likely enhanced the carbon combustion properties of Tl_2_O_3_. SEM observation and EDS mapping (Tl and Bi) of the Tl_2_O_3_‒95 wt% Bi_2_O_3_ powder confirmed that the particle size of the Tl_2_O_3_ and Bi_2_O_3_ powders was ~ 0.5 to 3 μm, which were homogeneously distributed (**Fig. ****12**).

#### *Carbon combustion characteristics of Tl*_*2*_*O*_*3*_*‒ x wt% Bi*_*2*_*O*_*3*_* (x* = *95‒99.5)*

**Figure 13:**
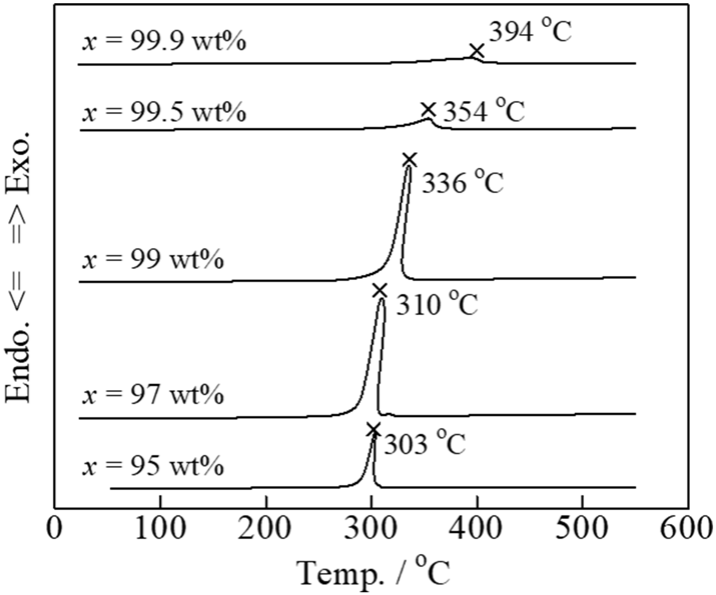
DSC curves of carbon black (2 wt%) mixed with the various Tl_2_O_3_‒ *x* wt%Bi_2_O_3_ (*x* = 95–99.9) composite catalysts.

Tl_2_O_3_ costs about ten times more than Bi_2_O_3_, thus the cost must be minimized to facilitate practical implementation. The carbon combustion performance was evaluated as the proportion of Bi_2_O_3_ was increased above 95 wt% (**Fig. ****13**). A large exothermal peak due to carbon combustion was observed up to 99 wt%, but the temperature was slightly higher at 336 °C. These findings indicated that the carbon combustion catalytic function of Tl_2_O_3_ was maintained at levels of only 1 wt%.

**Figure 14:**
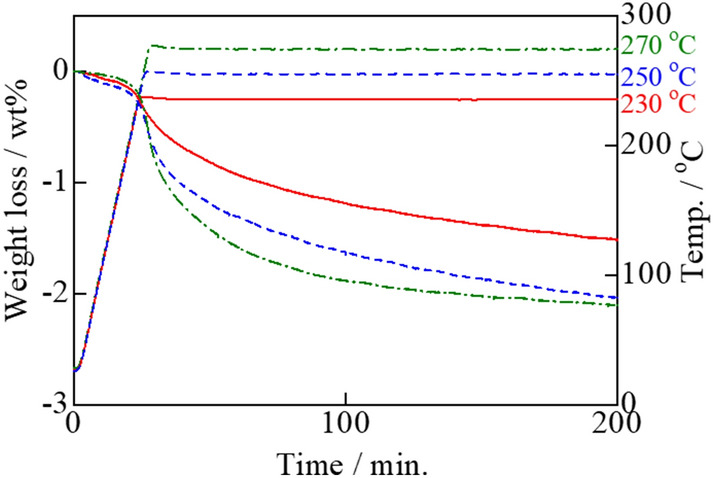
Isothermal TG analysis of carbon black (2 wt%) mixed with the Tl_2_O_3_‒95 wt%Bi_2_O_3_ composite catalyst at constant temperatures of 230, 250, and 270 °C for 3 h.

**Figure 15:**
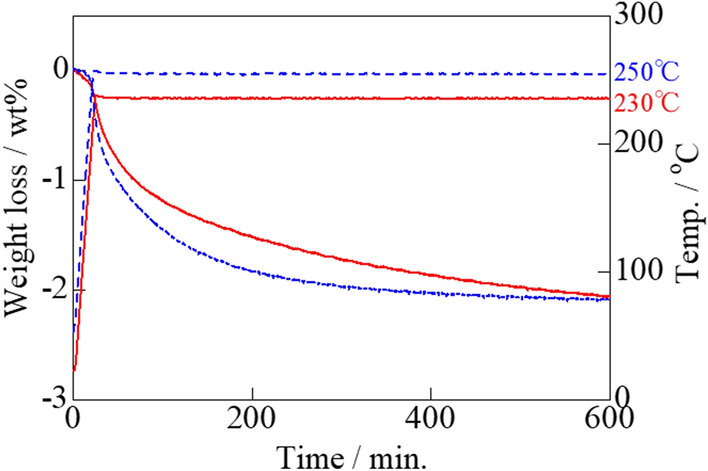
Isothermal TG analysis of carbon black (2 wt%) mixed with the Tl_2_O_3_‒95 wt% Bi_2_O_3_ composite catalyst at constant temperatures of 230 and 250 °C for 10 h.

Actual carbon combustion is assumed to begin at the rising temperature of the exothermal peak. The temperature at which real carbon combustion initiated and progressed was determined based on isothermal TG analysis of the Tl_2_O_3_‒95 wt% Bi_2_O_3_ composite catalyst. The sample was heated in an air stream to either 230, 250, or 270 °C, where the weight loss was measured as the sample was held at the respective temperature. The amount of carbon added was 2 wt%, thus the weight loss of 2 wt% after 3 h at 270 °C corresponded to the combustion of all added carbon (**Fig. ****14**). Further, a 2 wt% weight loss was confirmed at 10 h when heated at 230 and 250 °C (**Fig. ****15**). However, only a ~ 1 wt% weight loss was achieved after heating at 200 °C for 20 h. These results indicated that the carbon combustion reaction occurred at temperatures higher than ~ 230 °C.

### Carbon combustion using an alumina filter coated with Tl_2_O_3_‒95wt%Bi_2_O_3_

**Figure 16:**
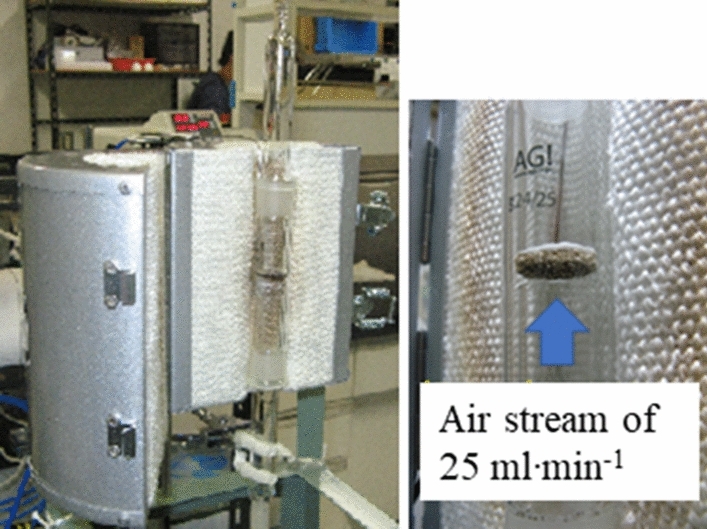
Experimental set-up for evaluating the carbon combustion characteristics of a porous alumina filter coated by the carbon combustion catalyst.

**Figure 17:**
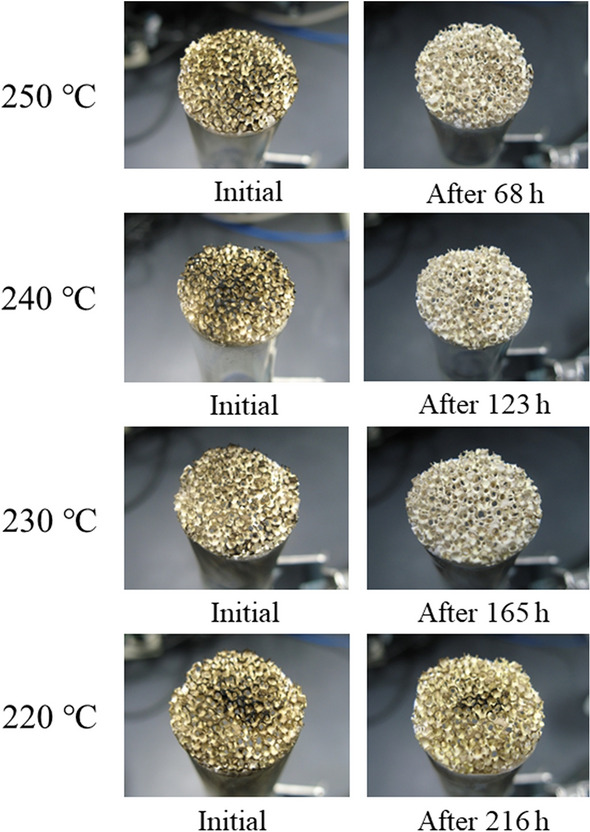
Carbon combustion using the coated porous alumina filter at 220 to 250 °C in an air stream of 25 ml‧min^-1^.

The combustion of carbon on a catalyst-supported alumina filter was demonstrated experimentally using the set-up shown in **Fig. ****16**. Carbon in the form of candle soot was deposited onto the catalyst-supported alumina filter, and the filter was held at constant temperatures of 250, 240, 230, and 220 °C (**Fig. ****17**). Complete removal of the soot via carbon combustion required ~ 68, ~ 123, and ~ 165 h at 250, 240, and 230 °C, respectively. The soot was not be completely removed at 220 °C after 216 h.

These results demonstrated that PM2.5 and DPM in exhaust gas could potentially be collected and decomposed using porous ceramic filters coated with the low-temperature PM combustion Tl_2_O_3_‒Bi_2_O_3_ catalytic system proposed in this study. This is expected to lead to the development of self-cleaning PM filters capable of decomposing and eliminating PM2.5 and DPM simply using the heat of the exhaust gas from a factory flue-gas stack or diesel vehicle muffler.

## Conclusions

The carbon combustion temperature of 660 °C can be reduced to 300 °C due to the catalytic effect of Tl_2_O_3_. This excellent carbon combustion catalytic property of Tl_2_O_3_ was further improved by mixing Tl_2_O_3_ with various oxides. Further, a porous ceramic filter was coated with the proposed Tl_2_O_3_-system catalyst to demonstrate its promising catalytic performance. Based on the findings of this study, the following conclusions can be drawn:

1. DSC analysis of the Tl_2_O_3_‒80wt% added oxide composite catalysts in the presence of 2 wt% carbon revealed that carbon combustion was enhanced by the addition of Bi_2_O_3_, Pr_6_O_11_, (CeO_2_)_0.8_(Gd_2_O_3_)_0.2_, (Bi_2_O_3_)_0.75_(Y_2_O_3_)_0.25_, La_9.7_Si_6_O_26.55_, and Pr_4.8_Bi_1.2_O_11_, where Bi_2_O_3_ exhibited a particularly outstanding performance. However, some of the oxides, including CeO_2_, α-Al_2_O_3_, ZrO_2_, MnO_2_, TiO_2_, Cr_2_O_3_, (ZrO_2_)_0.92_(Y_2_O_3_)_0.08_, and YMnO_3_, compromised the carbon combustion properties of Tl_2_O_3_.
2. The temperature of the exothermal peak due to carbon combustion (2 wt% carbon) during DSC analysis of the Tl_2_O_3_‒*x* wt% Bi_2_O_3_ (*x* = 5‒95) composite catalysts was reduced compared to that of pure Tl_2_O_3_. The best performance was observed at *x* values of 70, 80, and 90 wt%, where the temperature of the heat generation peak for carbon combustion was below 300 °C. Furthermore, a large exothermal peak was even observed at an *x* value of 99 wt%, but the temperature was ~ 336 °C.
3. Isothermal TG analysis of the Tl_2_O_3_‒95 wt% Bi_2_O_3_ composite catalyst with 2 wt% carbon led to a weight loss of ~ 2 wt% at 230 °C and higher, which was indicative of complete carbon combustion. An alumina filter was coated with the Tl_2_O_3_‒95wt%Bi_2_O_3_ catalyst and exposed to carbon in the form of candle soot. Holding the coated filter at 220 to 250 °C confirmed that carbon was completely combusted at 230 °C and higher.

## Methods

### Sample preparation

#### *Tl*_*2*_*O*_*3*_* and various oxide mixtures*

Tl_2_O_3_ powder (2 g) was wet-mixed with various oxide powders (8 g), including CeO_2_, α-Al_2_O_3_, ZrO_2_, TiO_2_, Bi_2_O_3_, Pr_6_O_11_, Cr_2_O_3_, MnO_2_, (ZrO_2_)_0.92_(Y_2_O_3_)_0.08_, (Bi_2_O_3_)_0.75_(Y_2_O_3_)_0.25_, (CeO_2_)_0.8_(Gd_2_O_3_)_0.2_, La_9.7_Si_6_O_26.55_, Pr_4.8_Bi_1.2_O_11_, or YMnO_3_, in deionized water using a planetary ball mill (Fritsch Co., Pulverisette 6) for 2 h. The mixtures were dried at 100 °C to obtain composites comprising Tl_2_O_3_–80 wt% added oxide.

#### *Tl*_*2*_*O*_*3*_* and Bi*_*2*_*O*_*3*_* mixture*

Mixtures of Tl_2_O_3_ with varying Bi_2_O_3_ contents (0.5‒99.9 wt%) were prepared from Tl_2_O_3_ (Kojundo Chemical Lab. Co., Ltd., 99.9% purity) and Bi_2_O_3_ (Kisan Kinzoku Chemicals Co.,Ltd., 99.9% purity) powders. The Tl_2_O_3_ and Bi_2_O_3_ powder mixtures (10 g) were wet-mixed in deionized water using a planetary ball mill for 3 h and dried at 100 °C to give composites comprising Tl_2_O_3_–*x* wt% Bi_2_O_3_ (*x* = 0.5–99.9).

### Characterization

The Tl_2_O_3_–80 wt% added oxide powders were analyzed using X-ray diffraction (XRD, Rigaku Co., MiniFlex II) in the 2θ range of 20° to 60° using CuK_α1_. The Tl_2_O_3_‒95 wt% Bi_2_O_3_ powder was observed and analyzed using scanning electron microscopy (SEM, Jeol Ltd., JSM-6510LA) with an energy dispersive spectroscopy (EDS) detector (Jeol Ltd., JED-2300).

The carbon combustion characteristics were evaluated by adding 2 wt% carbon black (Tokai Carbon Co., Ltd., Toka Black # 8500/F; average particle size = 14 nm; N_2_ adsorption specific surface area = 290 m^2^·g^-1^) to the Tl_2_O_3_–80 wt% added oxide powders and mixing thoroughly in an agate mortar for 1 to 2 min. The mixture (10 mg) was transferred to a platinum pan for differential scanning calorimetry (DSC) analysis (DSC8230; Rigaku Co.) between room temperature and 600 °C at a heating rate of 10 °C ·min^-1^ in a 20 mL ·min^-1^ air stream. The temperature of the DSC exothermic peak was used as the carbon combustion temperature. A mixture of 2 wt% carbon and Tl_2_O_3_‒95 wt%Bi_2_O_3_ powder was prepared in the same manner, and thermogravimetric (TG) analysis (TG8120; Rigaku Co.) was conducted under isothermal conditions for 3 to 20 h at 200, 230, 250, and 270 °C at a heating rate of 10 °C ·min^-1^ in a 20 mL ·min^-1^ air stream.

A ceramic filter coated with the Tl_2_O_3_‒95 wt% Bi_2_O_3_ composite catalyst was prepared. The Tl_2_O_3_‒95wt%Bi_2_O_3_ powder was dispersed in deionized water and coated on a porous Al_2_O_3_ ceramic foam filter (Shinagawa Fine Ceramics Co., Ltd., number of cells: 25–30 pieces/inch, φ20mm × t3mm) via a dipping method. The filter was fixed on a glass tube using an inorganic adhesive, and soot was deposited on the outer surface of the filter using a candle flame. The filter was transferred to a mantle heater furnace to evaluate its carbon decomposition and elimination performance. The condition of the soot deposited on the filter was observed every few hours under an air stream of 25 mL ‧min^-1^ from 200 to 270 °C provided by an air pump.
